# Comparative metabolome variation in *Brassica juncea* different organs from two varieties as analyzed using SPME and GCMS techniques coupled to chemometrics

**DOI:** 10.1038/s41598-024-69865-8

**Published:** 2024-08-27

**Authors:** Mohamed A. Farag, Vinod Goyal, Mostafa H. Baky

**Affiliations:** 1 Pharmacognosy Department, College of Pharmacy, Cairo University, Cairo, 11562 Egypt; 2 Department of Botany & Plant Physiology, CCS Haryana Agricultural University, Hisar, Haryana 125004 India; 3 Pharmacognosy Department, Faculty of Pharmacy, Egyptian Russian University, Badr City, 11829 Cairo Egypt

**Keywords:** Indian mustard, *Brassica juncea*, Brassicaceae, Nutrient metabolome, GC–MS, Chemometric analysis, Plant sciences, Climate sciences, Chemistry

## Abstract

Indian mustard (*Brassica juncea*; Brassicaceae) is an edible, oilseeds-yielding crop widely consumed as a food spice owing to its richness in nutrients with several health benefits. The current study aims to dissect the *B. juncea* metabolome heterogeneity among its different organs including leaf, stem, flower, and seed. Moreover, assessing the metabolome differences between two different varieties RH-725 and RH-761 grown at the same conditions. Gas chromatography-mass spectrometry (GC–MS) post-silylation was used to dissect the composition of nutrient metabolites coupled to multivariate data analysis. Variation in sulphur aglycones was measured using headspace-solid phase-microextraction HS-SPME coupled to GC–MS. A total of 101 nutrient metabolites were identified with the abundance of sugars represented by monosaccharides in all organs, except for seeds which were enriched in disaccharides (sucrose). α-Linolenic acid was detected as a marker fatty acid in leaf from RH-725 at 12.5 µg/mg. Malic acid was detected as a significant variant metabolite between the two varieties as detected in the leaf from the RH-725 variety at *ca.* 128.2 µg/mg compared to traces in RH-761. 7 Volatile sulphur compounds were detected at comparable levels in RH-725 and RH-761, with 3-butenyl isothiocyanate was the most abundant at 0.8–2 ng/mg.

## Introduction

Brassicaceae also known as cruciferous crops are a common food spices widely consumed worldwide owing to its nutritional and medicinal value^1^. Rapeseed mustard is a major oil-producing seed in *Brassica* crops belonging to family Brassicaceae^2^. Indian mustard (*Brassica juncea* L. Czern & Coss.) is an important edible, oil-yielding crop covering about 90% of the cultivated area under brassica oilseeds in India^3^. Mustard is the third-largest source of vegetable oil in the world, after soybeans and palm oil^3^. Owing to its richness in micronutrient (vitamins, minerals) and bioactive metabolites (glucosinolates and polyphenolics) with potential health benefits, Indian mustard (*Brassica juncea*) is commonly consumed as a food spice in several diets^2^. Glucosinolates are important sulphur-containing phytochemicals enriched in brassica vegetables and to account for their unique sharp hot and pungent flavor and taste^1^. Owing to its richness in nutrients and phytochemicals i.e. glucosinolates, several biological activities were reported in mustard including anti-inflammatory, antioxidant, cholesterol lowering, anti-obesity, anticancer, and antibacterial^2^.

*B. juncea* has the potential for quicker seed germination, high productivity, and heat and drought tolerance, along with enhanced insect and disease resistance^2,4^. There is a need to expand on the production of food crops but, on the other hand, environmental stresses (biotic and abiotic) can negatively affect the overall yield of agricultural crops^5^. Drought stress is recognized as the main factor leading to decline in agricultural productivity, because water shortage is persistently related to other major abiotic stresses, such as high-temperature stress and salinity^6^. The Indian Mustard (*B. juncea* L.) varieties are being breed for specific purposes based on the environmental factors particularly for abiotic stresses^7^. Among abiotic stresses, water shortage is quite prevalent in the northern regions of India in which only source of water for irrigation lies on rainwater. The most popular and latest released mustard varieties include RH-725 and RH-761 grown in Northern India, and their detailed data were listed in Table S1. They are the national released varieties for ‘High seed and oil yield under rain fed condition with wider adaptability’ in year 2019 (Statutory Order (S.O.) 3220 (E) Dated 06.09.2019) and 2018 (S.O. 1379 (E) Dated 27.03.2018), respectively for timely sown and rain fed conditions in Haryana, Punjab, Delhi, Jammu and Northern Rajasthan by the Ministry of Agriculture and Farmers' Welfare, Government of India.

Recently, different metabolomics technologies were applied for metabolites profiling in foodstuff either in targeted or untargeted manner to ensure quality and assess variation among food sources or genotypes^8^. Gas chromatography coupled to mass spectrometry (GC–MS) is a well adopted analytical tool for profiling of nutrient metabolites post silylation^9^ that has extensively ben reported in plants metabolomics studies and more towards food applications^10^. Considering the complexity of metabolites datasets, chemometric analysis is typically employed for analyzing data set generated from MS-based analysis and assess variation between different samples using unsupervised principal component analysis (PCA) and supervised orthogonal projection to least squares discriminant analysis (OPLS-DA)^11^. As *B. juncea* is valued for its unique taste and aroma, analysis of aroma compounds across its accessions ought to be examined as typical in brassica vegetables^12^.

The current work aims to present the first comparative GC–MS based profiling approach of nutrients and sulphur compounds in *Brassica juncea* L. (Indian mustard) in context to its different organs including seed, leaf, flower, and stem represented by it two varieties RH 725 and RH 761. Such results aid in future valorization approaches of its unused parts based on such detailed chemical makeup. Moreover, quantification of metabolites among different organs from the two varieties is presented to aid future cultivation approach targeting certain component yield or for its standardization.

## Results and discussion

The main goal of the current study was to assess metabolites heterogeneity in four organs derived from two different varieties in Indian mustard (*Brassica juncea*) in the context of their nutrient and sulfur aroma profiles using GC–MS-based metabolomics approach. Seed, leaf, flower, and stem of *B. juncea* obtained from the two varieties (RH-725 and RH-761) were included for comparative metabolites profiling analysed using chemometric tools.

### Primary metabolites profiling in *B. juncea* organs via GC–MS analysis (post-silylation)

**Figure 1:**
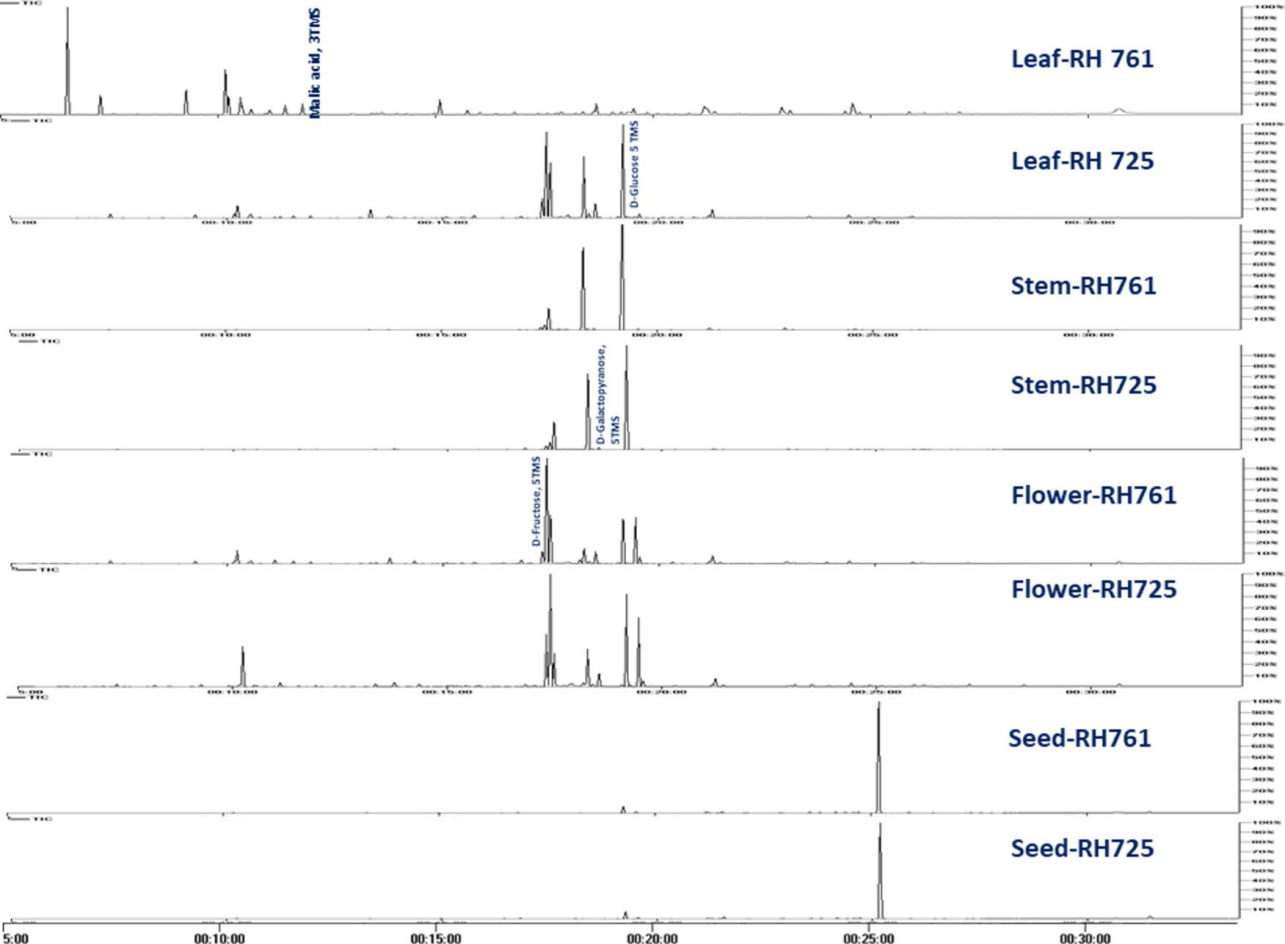
Representative GC–MS chromatograms of identified primary metabolites in *Brassica juncea* organs from different varieties post-silylation.

To assess variation in primary nutritive metabolites among *B. juncea* plant parts from two different varieties, GC–MS analysis was employed post-silylation. A total of 101 peaks (Table 1, Fig. 1) were annotated, including sugars (mono- and disaccharides), sugar alcohols, sugar acid, fatty acids/esters, organic acids, amino acids, nitrogenous compounds, sterols, phenolics and alcohols.

**Table 1:** Quantification of silylated metabolites in *Brassica juncea* organs from two different varieties (µg/mg) analyzed using GC–MS, n = 3.

Peak no	Average Rt (min)	Average RI	Metabolite name	Leaf, RH 761	Leaf, RH 725	Stem, RH761	Stem, RH725	Flower, RH761	Flower, RH725	Seed, RH725	Seed, RH761
61	20.824	2154	Phytol, TMS	0.06 ± 0.02	0.32 ± 0.07	0.00 ± 0.00	0.01 ± 0.00	0.01 ± 0.00	0.02 ± 0.01	0.01 ± 0.00	0.00 ± 0.00
Total alcohols				0.06 ± 0.02	0.32 ± 0.07	0.00 ± 0.00	0.01 ± 0.00	0.01 ± 0.00	0.02 ± 0.01	0.01 ± 0.00	0.00 ± 0.00
2	7.311	1098	l-Alanine, 2TMS	3.46 ± 0.22	4.36 ± 1.49	0.45 ± 0.16	0.33 ± 0.04	0.76 ± 0.21	1.36 ± 0.39	0.09 ± 0.01	0.02 ± 0.00
3	7.607	1115	Glycine, di-TMS	0.19 ± 0.08	0.26 ± 0.10	0.01 ± 0.01	0.01 ± 0.01	0.06 ± 0.01	0.10 ± 04	0.00 ± 0.00	0.00 ± 0.00
4	9.276	1211	l-Valine, 2TMS	3.74 ± 0.51	2.81 ± 0.69	0.16 ± 0.06	0.10 ± 0.01	0.54 ± 0.16	1.04 ± 0.18	0.06 ± 0.01	0.01 ± 0.00
5	10.178	1269	l-Leucine, 2TMS	5.90 ± 1.18	3.46 ± 0.75	0.14 ± 0.05	0.07 ± 0.01	0.63 ± 0.21	1.16 ± 0.24	0.02 ± 0.00	0.00 ± 0.00
7	10.520	1291	l-Isoleucine, 2TMS	2.17 ± 0.35	1.31 ± 0.24	0.07 ± 0.03	0.04 ± 0.01	0.28 ± 0.10	0.48 ± 0.06	0.02 ± 0.00	0.00 ± 0.00
8	10.558	1294	l-Ornithine, 3TMS	0.94 ± 0.07	2.33 ± 0.42	0.06 ± 0.01	0.11 ± 0.01	0.63 ± 0.15	0.92 ± 0.46	0.06 ± 0.01	0.01 ± 0.00
9	10.565	1294	l-Proline, 2TMS	0.48 ± 0.17	1.13 ± 0.24	0.01 ± 0.01	0.03 ± 0.01	0.14 ± 0.00	0.25 ± 0.12	0.00 ± 0.00	0.00 ± 0.00
13	11.550	1358	Serine, 3TMS	1.11 ± 0.09	1.45 ± 0.43	0.13 ± 0.03	0.14 ± 0.01	0.50 ± 0.12	0.67 ± 0.32	0.05 ± 0.00	0.01 ± 0.00
14	11.947	1384	l-Threonine, 3TMS	1.37 ± 0.11	1.54 ± 0.43	0.09 ± 0.03	0.08 ± 0.01	0.41 ± 0.13	0.59 ± 0.21	0.04 ± 0.00	0.01 ± 0.00
15	12.402	1416	Lanthionine, 4TMS	0.04 ± 0.01	0.13 ± 0.04	0.01 ± 0.01	0.01 ± 0.00	0.03 ± 0.02	0.08 ± 0.01	0.02 ± 0.00	0.01 ± 0.00
21	13.759	1518	l-5-Oxoproline, 2TMS	0.25 ± 0.03	1.44 ± 0.44	0.58 ± 0.25	0.52 ± 0.05	1.06 ± 0.20	1.87 ± 0.97	0.09 ± 0.01	0.07 ± 0.02
23	14.991	1613	L-Glutamic acid, 3TMS	0.15 ± 0.02	0.36 ± 0.12	0.03 ± 0.01	0.01 ± 0.00	0.08 ± 0.02	0.24 ± 0.13	0.43 ± 0.04	0.23 ± 0.06
24	15.102	1622	Phenylalanine, 2TMS	1.51 ± 0.25	0.93 ± 0.23	0.05 ± 0.02	0.03 ± 0.00	0.22 ± 0.07	0.45 ± 0.10	0.07 ± 0.00	0.03 ± 0.01
29	16.007	1698	l-Lysine, 3TMS	0.21 ± 0.11	0.06 ± 0.03	0.00 ± 0.00	0.00 ± 0.00	0.01 ± 0.00	0.01 ± 0.00	0.00 ± 0.00	0.00 ± 0.00
30	16.125	1710	l-Asparagine, 3TMS	0.01 ± 0.00	0.05 ± 0.02	0.05 ± 0.01	0.02 ± 0.01	0.01 ± 0.00	0.02 ± 0.00	0.11 ± 0.04	0.11 ± 0.03
32	16.822	1768	l-Glutamine, 3TMS	0.18 ± 0.03	1.00 ± 0.37	0.41 ± 0.25	0.63 ± 0.08	0.60 ± 0.16	1.12 ± 0.66	0.73 ± 0.09	0.42 ± 0.14
47	18.641	1935	l-Tyrosine, 3TMS	0.39 ± 0.12	0.69 ± 0.13	0.03 ± 0.02	0.02 ± 0.00	0.11 ± 0.04	0.23 ± 0.02	0.01 ± 0.00	0.00 ± 0.00
Total amino acids				22.11 ± 3.36	23.33 ± 6.17	2.28 ± 0.97	2.15 ± 0.26	6.08 ± 1.59	10.60 ± 3.92	1.80 ± 0.23	0.95 ± 0.27
51	19.331	2000	5,8,11-Eicosatrienoic acid, (Z)-, TMS	0.16 ± 0.06	1.13 ± 0.10	0.03 ± 0.01	0.03 ± 0.01	0.07 ± 0.02	0.08 ± 0.02	0.06 ± 0.01	0.04 ± 0.01
52	19.410	2007	Hexadecanoic acid, TMS	0.31 ± 0.02	1.04 ± 0.23	0.06 ± 0.02	0.05 ± 0.02	4.98 ± 2.78	0.73 ± 0.07	0.33 ± 0.06	0.14 ± 0.01
54	19.559	2024	Palmitic acid, TMS	2.52 ± 0.32	8.43 ± 0.48	2.75 ± 0.44	1.29 ± 0.18	4.68 ± 1.40	5.58 ± 1.31	7.02 ± 0.69	5.56 ± 0.59
62	21.147	2188	9,12-Octadecadienoic acid (Z,Z)-, TMS	0.41 ± 0.13	1.23 ± 0.32	0.02 ± 0.00	0.01 ± 0.00	0.05 ± 0.01	0.05 ± 0.03	0.01 ± 0.00	0.02 ± 0.00
63	21.177	2191	9-Octadecenoic acid, (E)-, TMS	1.50 ± 1.52	5.92 ± 1.31	2.90 ± 0.70	1.17 ± 0.22	2.98 ± 2.96	1.57 ± 0.48	1.88 ± 0.19	1.49 ± 0.09
64	21.228	2197	α-Linolenic acid, TMS	1.13 ± 0.74	12.59 ± 2.28	2.31 ± 0.58	0.83 ± 0.29	3.42 ± 1.26	4.36 ± 1.38	1.78 ± 0.18	1.73 ± 0.07
65	21.298	2204	Oleic acid, TMS	0.02 ± 0.00	0.53 ± 0.17	0.01 ± 0.00	0.02 ± 0.00	0.03 ± 0.01	0.16 ± 0.03	0.01 ± 0.00	0.01 ± 0.00
66	21.424	2218	Stearic acid, TMS	0.76 ± 0.56	1.20 ± 0.24	0.87 ± 0.09	0.40 ± 0.06	1.58 ± 0.98	1.33 ± 0.41	0.56 ± 0.04	0.40 ± 0.02
69	21.978	2280	9(E),11(E)- linoleic acid, TMS	0.13 ± 0.15	0.07 ± 0.00	0.09 ± 0.02	0.04 ± 0.00	0.11 ± 0.13	0.04 ± 0.00	0.06 ± 0.00	0.06 ± 0.01
70	21.984	2281	9,12-Octadecadienoic acid, TMS	0.02 ± 0.00	0.98 ± 0.39	0.02 ± 0.00	0.01 ± 0.00	0.05 ± 0.01	0.18 ± 0.03	0.01 ± 0.00	0.01 ± 0.00
71	22.895	2383	1-Monomyristin, 2TMS	0.01 ± 0.00	0.56 ± 0.22	0.02 ± 0.01	0.03 ± 0.00	0.05 ± 0.01	0.12 ± 0.03	0.02 ± 0.00	0.01 ± 0.00
72	22.958	2390	11-Eicosenoic acid,TMS	1.17 ± 1.30	1.26 ± 0.33	2.59 ± 0.56	1.00 ± 0.16	3.25 ± 2.94	1.56 ± 0.37	0.52 ± 0.05	0.41 ± 0.03
74	23.149	2413	Arachidic acid, TMS	0.66 ± 0.88	0.20 ± 0.04	1.21 ± 0.26	0.50 ± 0.11	1.46 ± 1.78	0.37 ± 0.07	0.22 ± 0.03	0.18 ± 0.01
76	24.149	2540	Octacosanoic acid, methyl ester	0.20 ± 0.05	1.04 ± 0.22	0.10 ± 0.03	0.05 ± 0.00	0.37 ± 0.07	0.57 ± 0.17	5.15 ± 1.61	3.96 ± 0.71
77	24.408	2573	1-Monopalmitin, 2TMS	2.90 ± 1.15	10.26 ± 4.89	1.60 ± 0.79	0.83 ± 0.39	1.87 ± 0.51	3.49 ± 1.20	2.52 ± 1.73	2.87 ± 1.45
78	24.577	2594	13-Docosenoic acid; TMS	1.77 ± 1.93	0.27 ± 0.06	1.43 ± 0.36	0.52 ± 0.10	1.29 ± 1.48	0.33 ± 0.06	1.40 ± 0.21	1.15 ± 0.09
80	24.746	2616	Behenic acid, TMS	0.36 ± 0.29	0.51 ± 0.07	0.66 ± 0.06	0.33 ± 0.04	0.67 ± 0.56	0.53 ± 0.28	4.43 ± 1.02	3.49 ± 0.62
82	24.910	2635	2-Trimethylsilyloxysebacic acid, 2TMS	0.28 ± 0.05	1.35 ± 0.33	0.03 ± 0.01	0.04 ± 0.02	0.05 ± 0.02	0.14 ± 0.04	0.02 ± 0.00	0.01 ± 0.00
85	25.766	2745	1-Monooleoylglycerol, 2TMS	0.03 ± 0.00	1.55 ± 0.56	0.21 ± 0.12	0.07 ± 0.02	0.10 ± 0.04	0.20 ± 0.12	0.02 ± 0.00	0.02 ± 0.00
86	25.875	2759	Glycerol monostearate, 2TMS	2.06 ± 0.62	6.15 ± 2.86	0.99 ± 0.49	0.52 ± 0.26	1.20 ± 0.22	2.12 ± 0.79	1.88 ± 1.25	2.24 ± 1.11
87	26.095	2787	15-Tetracosenoic acid, TMS	0.13 ± 0.11	0.13 ± 0.05	0.27 ± 0.05	0.12 ± 0.01	0.30 ± 0.21	0.24 ± 0.07	0.11 ± 0.02	0.09 ± 0.01
89	26.226	2802	Lignoceric acid, TMS	0.20 ± 0.22	0.26 ± 0.04	0.39 ± 0.06	0.17 ± 0.02	0.42 ± 0.39	0.19 ± 0.04	0.13 ± 0.00	0.10 ± 0.01
Total fatty acid/ester				16.73 ± 10.13	56.67 ± 15.23	18.57 ± 4.67	8.04 ± 1.94	28.98 ± 17.77	23.93 ± 7.03	28.14 ± 7.09	24.01 ± 4.86
25	15.247	1635	2,3,4,5-Tetrahydroxypentanoic acid-1,4-lactone, 3TMS	0.15 ± 0.02	0.42 ± 0.36	0.38 ± 0.13	0.07 ± 0.02	1.99 ± 0.85	3.85 ± 1.62	0.23 ± 0.04	0.18 ± 0.04
43	18.172	1891	Gluconolactone, 4TMS derivative	0.05 ± 0.01	0.46 ± 0.09	0.19 ± 0.10	0.20 ± 0.06	1.84 ± 0.66	3.07 ± 0.05	0.14 ± 0.00	0.09 ± 0.02
Total lactones				0.21 ± 0.04	0.88 ± 0.45	0.57 ± 0.22	0.27 ± 0.09	3.83 ± 1.51	6.92 ± 1.67	0.37 ± 0.04	0.27 ± 0.06
12	11.197	1335	Uracil, 2TMS	0.31 ± 0.02	0.01 ± 0.00	0.01 ± 0.00	0.01 ± 0.00	0.01 ± 0.00	0.01 ± 0.00	0.00 ± 0.00	0.00 ± 0.00
16	13.098	1469	2-Aminomalonic acid, 3TMS	0.15 ± 0.04	0.58 ± 0.19	0.03 ± 0.01	0.03 ± 0.00	0.08 ± 0.04	0.14 ± 0.02	0.02 ± 0.00	0.01 ± 0.00
18	13.461	1496	3-Amino-2-piperidone, 2TMS	0.03 ± 0.01	0.07 ± 0.02	0.01 ± 0.00	0.01 ± 0.00	0.12 ± 0.04	0.12 ± 0.05	0.05 ± 0.00	0.02 ± 0.00
40	17.894	1865	Adenine, 2TMS	0.26 ± 0.05	0.88 ± 0.20	0.05 ± 0.03	0.01 ± 0.00	0.14 ± 0.04	0.30 ± 0.05	0.00 ± 0.00	0.00 ± 0.00
60	20.541	2125	Guanine, TMS	0.05 ± 0.01	0.46 ± 0.23	0.01 ± 0.01	0.00 ± 0.00	0.02 ± 0.01	0.06 ± 0.02	0.00 ± 0.00	0.00 ± 0.00
73	23.087	2405	5-Methyluridine, 3TMS	0.09 ± 0.11	0.37 ± 0.14	0.18 ± 0.04	0.07 ± 0.01	0.12 ± 0.08	0.41 ± 0.25	0.06 ± 0.01	0.05 ± 0.01
83	24.965	2643	Adenosine, 4TMS	0.02 ± 0.02	0.10 ± 0.02	0.01 ± 0.00	0.05 ± 0.01	0.07 ± 0.02	0.19 ± 0.05	0.03 ± 0.00	0.02 ± 0.01
94	28.706	3026	Guanosine, N,N-dimethyl, 4TMS	0.00 ± 0.00	0.03 ± 0.01	0.00 ± 0.00	0.02 ± 0.00	0.00 ± 0.00	0.01 ± 0.00	0.37 ± 0.04	0.18 ± 0.05
Total nitrogenous				0.92 ± 0.26	2.50 ± 0.82	0.30 ± 0.10	0.19 ± 0.03	0.56 ± 0.24	1.24 ± 0.44	0.54 ± 0.05	0.30 ± 0.08
1	6.195	998	Lactic acid, 2TMS	0.57 ± 0.32	0.04 ± 0.01	0.03 ± 0.01	0.04 ± 0.01	0.14 ± 0.05	0.21 ± 0.08	0.04 ± 0.00	0.04 ± 0.01
10	10.771	1308	Butanedioic acid, 2TMS	11.00 ± 2.24	9.70 ± 1.30	2.06 ± 1.18	0.71 ± 0.12	0.51 ± 0.28	1.27 ± 0.53	0.51 ± 0.11	0.25 ± 0.04
17	13.332	1486	Malic acid, 3TMS	0.11 ± 0.07	128.25 ± 55.58	7.88 ± 5.04	4.83 ± 0.41	3.98 ± 1.41	11.64 ± 6.81	4.19 ± 0.61	3.93 ± 1.08
22	14.348	1563	2,3,4-Trihydroxybutyric acid, 4TMS	2.16 ± 0.27	7.95 ± 2.42	0.33 ± 0.13	0.17 ± 0.01	8.25 ± 2.79	15.35 ± 1.09	1.11 ± 0.20	0.28 ± 0.08
28	15.734	1676	Hexanoic acid, TMS	6.53 ± 1.02	29.01 ± 9.52	1.17 ± 0.46	0.89 ± 0.11	3.69 ± 1.71	6.33 ± 1.52	0.47 ± 0.03	0.24 ± 0.10
33	16.955	1779	Azelaic acid, 2TMS	0.58 ± 0.06	0.74 ± 0.21	0.05 ± 0.01	0.05 ± 0.01	0.16 ± 0.05	0.30 ± 0.02	0.03 ± 0.01	0.03 ± 0.00
34	17.044	1787	2,3,4-Trihydroxybutyric acid, 4TMS	0.27 ± 0.04	0.46 ± 0.09	1.33 ± 1.02	0.11 ± 0.01	3.48 ± 1.38	6.65 ± 2.43	0.15 ± 0.05	0.12 ± 0.00
Total organic acids				21.22 ± 4.02	176.16 ± 69.14	12.86 ± 7.84	6.79 ± 0.69	20.21 ± 7.67	41.74 ± 12.48	6.50 ± 1.01	4.88 ± 1.33
56	19.865	2056	Sinapinic acid, 2TMS	0.01 ± 0.00	0.01 ± 0.00	0.01 ± 0.00	0.01 ± 0.00	0.01 ± 0.00	0.01 ± 0.00	0.16 ± 0.01	0.12 ± 0.03
59	20.330	2103	Cinnamic acid, 3,5-dimethoxy-4-(trimethylsiloxy)-, methyl ester	0.01 ± 0.00	0.11 ± 0.05	0.01 ± 0.00	0.01 ± 0.00	0.04 ± 0.02	0.16 ± 0.09	0.02 ± 0.00	0.01 ± 0.00
67	21.544	2231	Sinapinic acid, 2TMS	0.03 ± 0.02	0.18 ± 0.15	0.02 ± 0.01	0.02 ± 0.00	0.04 ± 0.02	0.05 ± 0.02	2.87 ± 0.29	1.99 ± 0.42
68	21.810	2260	Catechin-, 5TMS	0.01 ± 0.00	0.24 ± 0.09	0.01 ± 0.00	0.01 ± 0.00	0.02 ± 0.00	0.06 ± 0.01	0.01 ± 0.00	0.01 ± 0.00
92	27.534	2920	γ.-Tocopherol, TMS	0.02 ± 0.02	0.02 ± 0.01	0.01 ± 0.00	0.01 ± 0.00	0.04 ± 0.04	0.05 ± 0.02	0.55 ± 0.05	0.52 ± 0.04
95	29.374	3086	4-Hydroxy-3-methoxyphenylglycol, 3TMS	0.01 ± 0.00	0.11 ± 0.04	0.01 ± 0.00	0.01 ± 0.00	0.01 ± 0.00	0.02 ± 0.01	0.43 ± 0.05	0.26 ± 0.08
Total phenolics				0.08 ± 0.05	0.66 ± 0.34	0.07 ± 0.01	0.06 ± 0.01	0.16 ± 0.08	0.35 ± 0.14	4.04 ± 0.40	2.90 ± 0.57
96	29.810	3125	Campesterol, TMS	0.06 ± 0.01	0.14 ± 0.04	0.12 ± 0.05	0.09 ± 0.03	0.22 ± 0.04	0.23 ± 0.11	1.03 ± 0.08	0.79 ± 0.17
98	30.647	3200	β-Sitosterol, TMS	0.57 ± 0.13	0.83 ± 0.09	0.29 ± 0.13	0.19 ± 0.07	0.88 ± 0.26	1.08 ± 0.49	1.38 ± 0.09	1.28 ± 0.21
Total sterols				0.64 ± 0.14	0.97 ± 0.13	0.41 ± 0.17	0.29 ± 0.11	1.10 ± 0.30	1.31 ± 0.60	2.41 ± 0.17	2.07 ± 0.38
11	11.116	1330	Glyceric acid, 3TMS	0.36 ± 0.04	1.48 ± 0.49	0.18 ± 0.05	0.09 ± 0.00	1.43 ± 0.57	2.82 ± 0.47	0.02 ± 0.00	0.01 ± 0.00
53	19.458	2013	d-Gluconic acid, 6TMS	0.20 ± 0.18	2.37 ± 0.58	0.11 ± 0.04	0.06 ± 0.00	13.46 ± 8.11	30.31 ± 8.22	0.22 ± 0.02	0.15 ± 0.04
55	19.744	2043	α.-d-Glucopyranuronic acid, 5TMS	0.03 ± 0.00	0.13 ± 0.04	0.02 ± 0.01	0.01 ± 0.00	0.04 ± 0.01	0.10 ± 0.01	0.01 ± 0.00	0.01 ± 0.00
57	20.127	2083	Galacturonic acid, 4TMS	0.00 ± 0.00	0.13 ± 0.04	0.00 ± 0.00	0.00 ± 0.00	0.01 ± 0.00	0.02 ± 0.00	0.01 ± 0.00	0.01 ± 0.00
Total sugar acids				0.60 ± 0.23	4.10 ± 1.15	0.31 ± 0.11	0.17 ± 0.01	14.94 ± 8.69	33.24 ± 8.70	0.26 ± 0.02	0.18 ± 0.05
6	10.249	1274	Glycerol, 3TMS	4.74 ± 0.61	18.32 ± 6.48	1.04 ± 0.33	0.33 ± 0.05	4.75 ± 0.92	15.91 ± 2.59	0.84 ± 0.12	0.57 ± 0.08
19	13.550	1503	Erythritol, 4TMS	0.13 ± 0.02	0.01 ± 0.00	0.00 ± 0.00	0.00 ± 0.00	0.01 ± 0.00	0.01 ± 0.00	0.01 ± 0.00	0.00 ± 0.00
20	13.655	1511	Threitol, 4TMS	0.33 ± 0.03	0.04 ± 0.01	0.02 ± 0.01	0.01 ± 0.01	0.03 ± 0.00	0.06 ± 0.03	0.01 ± 0.00	0.01 ± 0.00
27	15.665	1669	Ribitol, 5TMS	0.01 ± 0.00	0.12 ± 0.04	0.10 ± 0.04	0.02ر0.00	0.02 ± 0.00	0.06 ± 0.01	0.01 ± 0.00	0.01 ± 0.00
48	18.746	1945	d-Glucitol, 6TMS	4.01 ± 2.41	0.04 ± 0.01	0.02 ± 0.01	0.02 ± 0.00	0.03 ± 0.01	0.04 ± 0.01	0.23 ± 0.01	0.19 ± 0.03
58	20.318	2102	Myo-Inositol, 6TMS	0.20 ± 0.06	1.15 ± 0.56	0.78 ± 0.52	0.33 ± 0.13	0.40 ± 0.35	0.58 ± 0.04	0.20 ± 0.02	0.10 ± 0.01
Total sugar alcohol				9.41 ± 3.13	19.69 ± 7.10	1.95 ± 0.90	0.72 ± 0.19	5.23 ± 1.29	16.65 ± 2.68	1.30 ± 0.16	0.87 ± 0.13
26	15.441	1651	d-Arabinose, 4TMS	0.06 ± 0.03	1.12 ± 0.35	0.40 ± 0.12	0.08 ± 0.01	0.42 ± 0.10	0.79 ± 0.13	0.02 ± 0.00	0.01 ± .01
31	16.417	1733	d-Fructose, 5TMS	0.30 ± 0.04	0.61 ± 0.11	0.08 ± 0.01	0.35 ± 0.04	0.45 ± 0.07	0.20 ± 0.01	0.74 ± 0.08	0.43 ± 0.12
35	17.184	1798	d-Erythrotetrofuranose, 3TMS	0.01 ± 0.00	0.10 ± 0.02	0.12 ± 0.02	0.06 ± 0.04	0.02 ± 0.00	0.02 ± 0.01	0.01 ± 0.00	0.00 ± 0.00
36	17.494	1828	d-Fructose, 5TMS	0.08 ± 0.01	62.03 ± 9.05	30.36 ± 10.60	30.07 ± 6.37	31.08 ± 25.58	38.54 ± 16.45	0.09 ± 0.01	0.06 ± 0.01
37	17.732	1850	Arabinofuranose, 4TMS	0.20 ± 0.05	1.61 ± 0.31	1.44 ± 0.13	0.71 ± 0.19	0.32 ± 0.06	0.53 ± 0.15	0.03 ± 0.02	0.02 ± 0.00
38	17.790	1855	d-Galactofuranoside, 4TMS	0.25 ± 0.05	0.60 ± 0.10	1.19 ± 0.46	0.38 ± 0.08	0.41 ± 0.19	0.24 ± 0.37	0.05 ± 0.01	0.04 ± 0.01
39	17.842	1860	l-Sorbopyranose, 5TMS	0.25 ± 0.05	1.26 ± 0.31	0.52 ± 0.12	0.39 ± 0.06	0.54 ± 0.07	1.20 ± 0.17	0.04 ± 0.03	0.02 ± 0.02
41	17.904	1866	Xylose, 4TMS	0.46 ± 0.08	3.94 ± 1.00	1.14 ± 0.28	0.47 ± 0.04	0.82 ± 0.14	1.68 ± 0.12	0.01 ± 0.00	0.01 ± 0.00
42	18.116	1884	d-(-)-Ribofuranose, 4TMS (isomer 1)	0.05 ± 0.01	0.44 ± 0.04	0.05 ± 0.01	0.10 ± 0.09	1.84 ± 0.65	2.05 ± 1.75	0.92 ± 0.32	0.23 ± 0.06
44	18.228	1896	d-Fructose, 5TMS	0.06 ± 0.01	0.57 ± 0.10	0.32 ± 0.15	0.33 ± 0.08	0.10 ± 0.01	0.30 ± 0.13	0.01 ± 0.00	0.00 ± 0.00
45	18.274	1901	d-Galactopyranose, 5TMS	0.16 ± 0.11	60.08 ± 11.13	97.59 ± 11.06	69.67 ± 4.36	10.54 ± 2.49	32.68 ± 21.24	0.67 ± 0.11	0.35 ± 0.06
46	18.387	1911	α.-d-Glucopyranose, 5TMS	0.61 ± 0.13	6.96 ± 1.85	2.75 ± 0.61	1.08 ± 0.02	1.01 ± 0.17	1.73 ± 0.05	0.01 ± 0.00	0.01 ± 0.00
49	19.065	1975	Arabinofuranose, 4TMS	0.52 ± 0.06	1.92 ± 0.50	0.11 ± 0.08	0.0 ± 0.04	0.52 ± 0.03	1.02 ± 0.09	0.31 ± 0.04	0.12 ± 0.02
50	19.177	1986	d-Glucose 5 TMS	0.09 ± 0.02	98.11 ± 9.84	126.45 ± 11.76	93.39 ± 5.87	26.70 ± 3.28	63.68 ± 24.92	0.12 ± 0.01	7.41 ± 1.32
75	23.883	2506	Methyl galactoside, 4TMS	0.01 ± 0.00	0.16 ± 0.12	0.04 ± 0.03	0.11 ± 0.01	0.50 ± 0.24	0.72 ± 0.45	0.01 ± 0.00	0.01 ± 0.00
79	24.707	2610	Sucrose, 8TMS	0.09 ± 0.10	0.07 ± 0.02	0.18 ± 0.02	0.09 ± 0.01	0.15 ± 0.18	0.13 ± 0.11	1.20 ± 0.28	0.95 ± 0.17
81	24.838	2627	Unknown	0.05 ± 0.01	0.27 ± 0.05	0.17 ± 0.12	0.14 ± 0.06	0.09 ± 0.04	0.27 ± 0.17	0.01 ± 0.00	0.00 ± 0.00
84	25.163	2669	Sucrose, 8TMS	0.07 ± 0.10	0.13 ± 0.05	0.01 ± 0.00	0.01 ± 0.01	0.02 ± 0.00	0.03 ± 0.02	133.02 ± 6.25	98.69 ± 16.98
88	26.12	2790	Unknown	0.05 ± 0.05	0.32 ± 0.12	0.18 ± 0.09	0.05 ± 0.02	0.44 ± 0.15	0.63 ± 0.25	0.05 ± 0.00	0.05 ± 0.00
90	26.718	2846	Melibiose, 8TMS	0.20 ± 0.03	0.41 ± 0.16	0.08 ± 0.04	0.03 ± 0.00	0.02 ± 0.01	0.05 ± 0.01	0.11 ± 0.01	0.09 ± 0.02
91	27.169	2887	Trehalose, 8TMS	0.08 ± 0.02	0.09 ± 0.10	0.01 ± 0.01	0.01 ± 0.00	0.31 ± 0.09	0.79 ± 0.30	0.04 ± 0.02	0.06 ± 0.02
93	28.429	3000	Unknown	0.03 ± 0.02	0.04 ± 0.01	0.02 ± 0.00	0.02 ± 0.00	0.12 ± 0.07	0.36 ± 0.35	0.02 ± 0.00	0.03 ± 0.01
97	30.099	3151	Sucrose, 8TMS	0.02 ± 0.01	0.03 ± 0.01	0.02 ± 0.00	0.02 ± 0.00	0.02 ± 0.00	0.04 ± 0.00	0.27 ± 0.05	0.15 ± 0.03
99	30.708	3206	Maltotriose 8TMS	0.55 ± 0.12	0.01 ± 0.00	0.00 ± 0.00	0.00 ± 0.00	0.00 ± 0.00	0.01 ± 0.00	0.01 ± 0.00	0.54 ± 0.03
100	31.431	3271	Trehalose, 8TMS	0.03 ± 0.02	0.01 ± 0.00	0.00 ± 0.00	0.00 ± 0.00	0.00 ± 0.00	0.01 ± 0.00	2.90 ± 0.20	1.29 ± 0.36
101	31.966	3319	d-Raffinose, 8TMS	0.11 ± 0.07	0.02 ± 0.01	0.01 ± 0.00	0.00 ± 0.00	0.01 ± 0.00	0.01 ± 0.00	0.05 ± 0.07	0.13 ± 0.01
Total sugars				4.39 ± 1.21	240.89 ± 35.35	263.25 ± 35.72	197.65 ± 17.42	76.44 ± 33.63	147.70 ± 67.25	140.71 ± 7.53	110.69 ± 19.25

*Average Rt*(*min*) average retention time by minutes, *Average RI* average retention index.

### Sugars

Sugars were detected as the most dominant primary metabolites represented by 26 peaks identified in *B. juncea* different organs viz. leaf, stem, flower, and seed in both varieties. The highest levels of sugars represented by monosaccharides were detected in brassica stem RH-761 amounting for *ca.* 263.2 µg/mg, followed by leaf stem, and flower collected from RH-725 at 240.8, 197.65, and 147.7 µg/mg. In contrast, the highest levels of disaccharides were detected in seed from both varieties at 110–140 µg/mg, respectively. In contrast, the lowest sugar level was detected in the leaf RH-761 at *ca.* 4.3 µg/mg. Such variation in sugar levels indicate that *B. juncea* organs from the variety RH-725 are in general higher in sugar content, except only for the stem than RH-761. Glucose was the major sugar in leaf RH-725, stem and flower from both RH-761 and RH-725 detected at 26.7–126.4 µg/mg. Next to glucose, fructose and galactose were abundant in *B. juncea* leaf RH-725, stem and flower from both RH-761 and RH-725 at 30.3–62.03 and 10.5–97.5 µg/mg, respectively. Seeds from both varieties showed trace levels of glucose, fructose, and galactose, likely as storage organ to be richer in fatty acids. Interestingly, in seeds major sugar was represented by sucrose detected at much higher level in seeds with higher level in variety RH-725 at *ca.* 133.02 µg/mg compared to 98.6 µg/mg in RH-761. The abundance of sucrose in *B. juncea* seed pose it as the most palatable and edible part. Conclusively regarding sugar profile, organs from *B. juncea* variety RH-725 showed higher levels of monosaccharides than variety RH-761, except stem RH-761 that encompassed higher level of sugars among all organs. Among organs from both varieties, seeds contained higher levels of disaccharides. Free sugars such as stachyose, raffinose, melibiose, galactose, glucose and fructose were previously detected in *B. juncea*^13^.

Unlike free sugars, sugar alcohols were detected at much lower levels in *B. juncea* organs from different varieties being detected at comparable levels of 19.9 and 16.6 µg/mg in leaf and flower RH-725, respectively. Glycerol was detected as the most abundant sugar alcohol in leaf and flower for variety RH-725 at 18.3 and 15.9 µg/mg, respectively. Likewise, sugar acids were detected at high level in *B. juncea* flowers from the two varieties with higher abundance in variety RH-725 at *ca*. 33.2 compared to 14.9 µg/mg in RH-761. Gluconic acid, an important sugar acid used in food and pharmaceutical industry^14^ was detected as the most abundant sugar acid in brassica flower at *ca.* 30.3 and 13.4 µg/mg in RH-725 and RH-761, respectively. Retrieving sugars results and comparison among brassica organs from the two varieties revealed that variety RH-725 was the richest in free sugars and its derivatives represented by glucose, fructose, galactose, sucrose, glycerol, and gluconic acid.

### Amino acids/nitrogenous compounds/phenolics

Amino acids play a pivotal role in human health owing to their nutritional and medicinal importance in the regulation of anti-oxidative, metabolic, and immune responses^8^. Amino acids were represented by 17 peaks detected at comparable higher levels in leaf and flower from both varieties compared to other organs and detected at higher level in leaf RH-725 and flower RH-725 at *ca*. 23.3 and 10.6 µg/mg, respectively. Such high amino acids level in leaf and flower from RH-725 pose it for improved nutritional makeup compared variety RH-761. Alanine, valine, and leucine were the most abundant amino acids detected among brassica organs, specially leaf and flower form RH-725 variety. Compared to the food sources of amino acids including meat, milk, cheese, and grains, *B. juncea* leaf and flower are considered as important source for nutritionally important amino acids. Amino acids were previously reported in Indian mustard seeds at high levels including phenylalanine, tyrosine, methionine, cystine, leucine, valine, and lysine^15^.

Nitrogenous compounds were detected at trace levels in all examined organs ranging from 0.2–2.5 µg/mg and 0.3–0.9 µg/mg in organs from RH-725 and RH-761, respectively, with only leaf RH-725 and flower RH-725 that showed relatively higher levels. Likewise, phenolics were detected at trace levels in organs from the two varieties except a comparable level was detected in seed from RH-725 and RH-761 at *ca*. 3–4µg/mg, though it should be noted that GC/MS is not suited for profiling of phenolics considering their polar nature and warranting for using LC/MS technique^16^. Several polyphenols were previously reported in *B. juncea* using high-performance liquid chromatography (HPLC)^17^. Sinapinic acidwas the major phenolic acid in *B. juncea* seed specially in variety RH-725 (3.03 µg/mg). Sinapinic acid is a chief phenolic acid previously identified in rapeseed with potential health benefits including anti-inflammatory, hepatoprotective, cardioprotective, anti-cancer, and anti-diabetic^18^.

### Fatty acids/esters/sterols

Fatty acids/esters represented by 22 peaks were detected at relatively high levels in all organs of *B. juncea* with higher levels in leaf RH-725 at 56.6 µg/mg represented by saturated, monounsaturated and polyunsaturated fatty acids. α-Linolenic acid is a poly-unsaturated ω-3 fatty acid with positive effects on atherosclerosis incidence risk and hence reduce cardiovascular diseases risk^19^ found at high level in leaf RH-725 at 12.5 µg/mg. Palmitic acid was detected as the major saturated fatty acids in leaf RH-725 and seed RH-725 at 8.4 and 7.02 µg/mg, respectively. In a previous study, fatty acids were previously detected in *B. juncea* specially unsaturated fatty acids including oleic acid and palmitoleic acid^20^. Moreover, *B. juncea* oil was reported to contain a balanced levels of both saturated and unsaturated fatty acids specially both omega-6 and omega-3 polyunsaturated fatty acids^13^.

Regarding fatty acyl esters, 1-monopalmitin, an esterified product of palmitic acid previously identified in a variety of plant extracts and to exhibit antibacterial activities^19^, was detected at highest levels in leaf RH-725 of 10.2 µg/mg. Unlike fatty acids, sterols were detected at much lower levels in *B. juncea* represented mainly by two peaks identified as campesterol and *β*-sitosterol with relative high level in seed RH-725 at 2.4 µg/mg compared to other accessions. Such results indicated that *B. juncea* organs from variety RH-725 is the richest in fatty acids and sterols compared to that from RH-761.

### Organic acids/alcohols/lactones

Organic acids were detected in all organs from the two varieties at comparable levels, with highest level found in leaf RH-725 at 176 µg/mg followed by flower RH-725 at 41.7 µg/mg. Organic acids are important in food products as natural preservative and can promote food digestion as well as aiding in protein utilization^12^. Malic acid was detected as the major form in *B. juncea* leaf RH-725 at *ca.* 128.2 µg/mg. Malic acid exhibits potential antioxidant capacity asides from its action as food preservative^21^. Such high level of malic acid in leaf from the variety RH-725 could be a marker for genetic variation between the two varieties RH-725 and RH-761 which has yet to be confirmed using QTL sequencing. Malic acid was previously detected in *B. juncea* flower in a study done by El Majdoub et al.^22^. Next to malic acid, hexanoic acid, a short-chain monocarboxylic acid was detected at high level in leaf RH-725 29.01 µg/mg. Compared to acids, alcohols were detected at trace levels in most samples represented mainly by phytol, a diterpene alcohol with potential health benefits^23^. Likewise, lactones were detected at trace levels in all organs, except in flower of RH-725 at 6.9 µg/mg versus 3.8 µg/mg in RH-761. Tetrahydroxypentanoic acid-1,4-lactone and gluconolactone were the two identified lactones found most abundant in flower RH-725.

## HCA and PCA analysis of post-silylated primary metabolites from *B. juncea* organs from two different varieties

**Figure 2:**
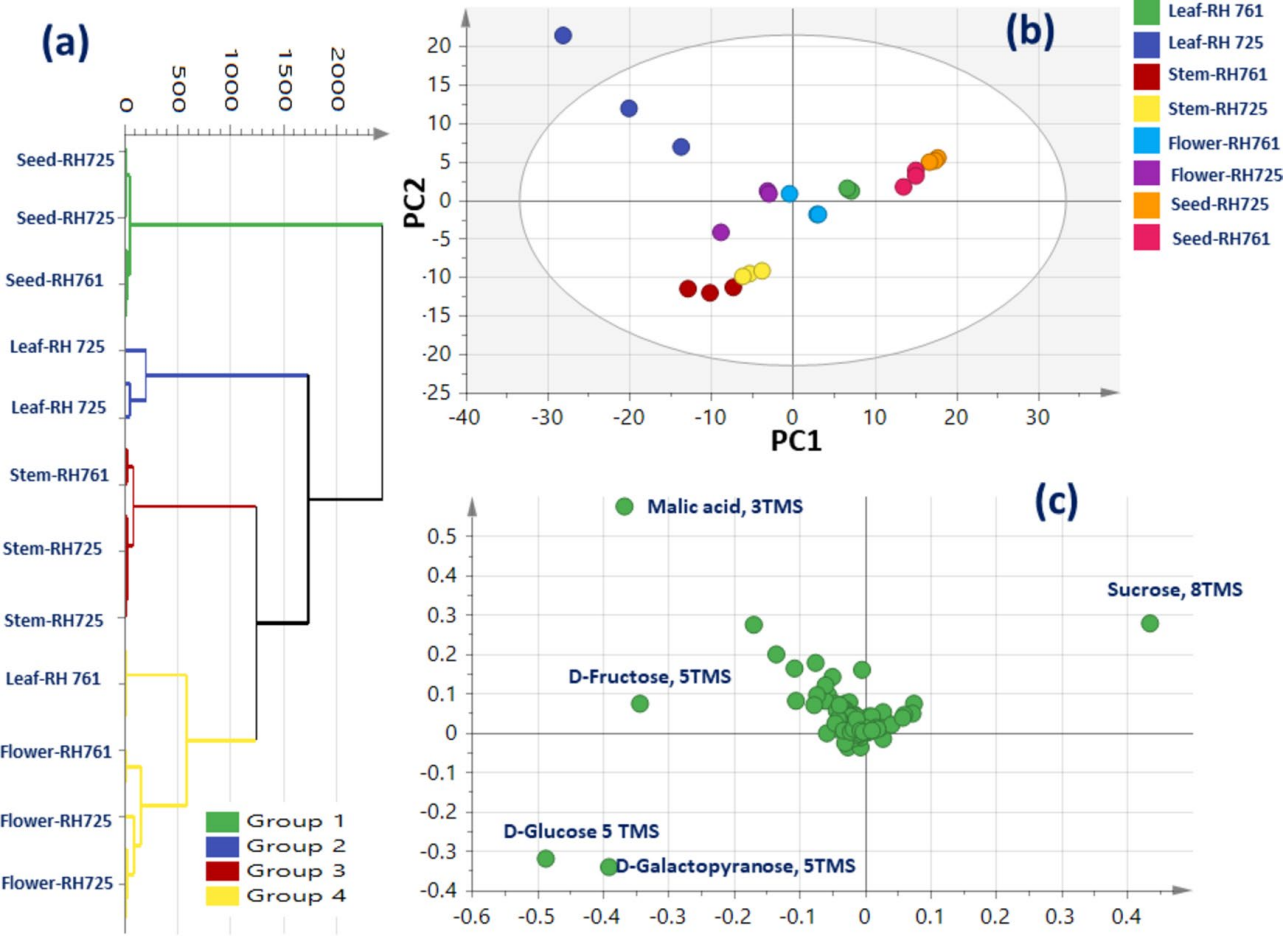
Unsupervised multivariate data analyses of *Brassica juncea* organs from different varieties derived from modeling of silylated primary metabolites dataset via GC–MS post silylation (n = 3). (**a**) HCA plot. (**b**) PCA score plot of PC1 vs. PC2 scores. (**c**) The respective loading plot for PC1 and PC2, providing mass peaks and their assignments. The metabolome clusters are placed in two-dimensional space at the distinct locations defined by two vectors of principal component PC1 = 51% and PC2 = 24%.

Unsupervised HCA and PCA analyses as multivariate data analysis tools were further employed to assess variations in primary metabolites among *B. juncea* organs (leaf, stem, flower, and seed) from two varieties (RH-725 and RH-761) (Table 1). HCA depicted a dendrogram of two distinct clusters (Fig. 2a), with seed from two varieties clustered separately in cluster 1, while other organs from the two varieties were divided in two subdivisions from cluster 2. Leaf RH-725 was clustered separately in sub cluster 2a. Moreover, stems from the two varieties were clustered against leaf-RH-761 and flower from the two varieties in subcluster 2b. However, clustering of organs from two different varieties together in cluster 2 indicated weak discrimination from HCA among varieties or even organs depending on silylated primary metabolites likely attributed to the weaker classification potential of primary compared to secondary metabolites^24^. A PCA model (Fig. 2b) prescribed by two orthogonal PCs accounted for 72% of the total variance, with segregation of leaf RH-725 alone at the positive left side of the score plot, whereas brassica seeds from two varieties were segregated together in the positive right side of PC1. Stems from both varieties were positioned towards the left side of PC1. Such unexpected clustering of leaf RH-725 specimens away from others is due to the large variations in metabolites content especially sugars, sugar alcohols, organic acids, fatty acid/esters, and amino acids. The corresponding loading plot (Fig. 2c) revealed for higher organic acids level in leaf RH-725 represented by malic acid and to account for its distant segregation. Moreover, the abundance of di-sugar i.e. sucrose (Peak 84) in seeds versus mono-sugar i.e. fructose, glucose, and galactose in other organs, suggestive that seeds are more rich in di sugars versus mono sugars in all other organs. PCA modelling results inferred that in particular leaf RH-725 and seeds (RH-725 and RH-761) were most distinguished from all other organs.

## OPLS-DA Analysis of *B. juncea* organs from the two different varieties

**Figure 3:**
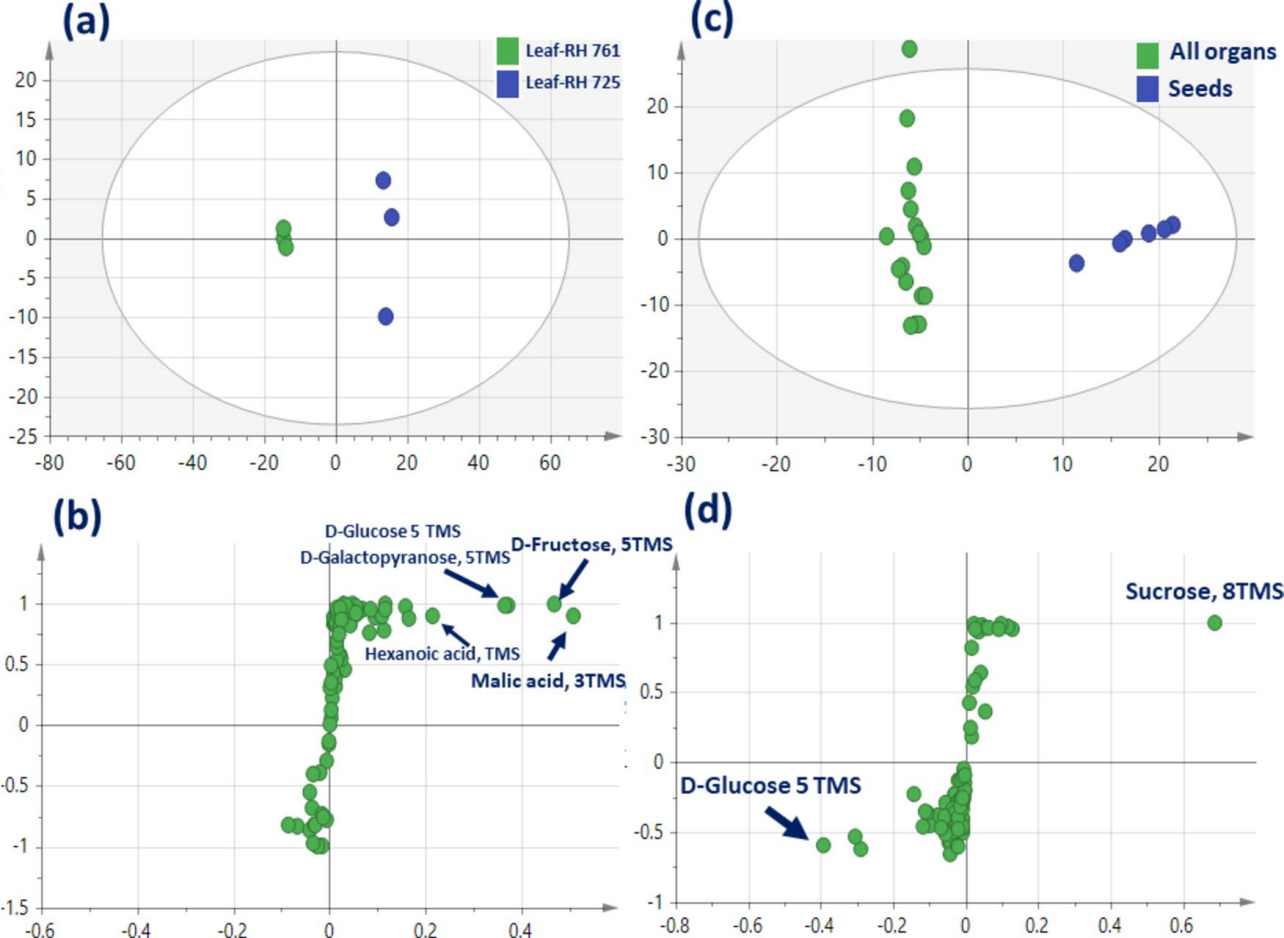
GC–MS-based OPLS-DA score plot (**a**) derived from modeling silylated primary metabolites of *Brassica juncea* leaf-RH 761 versus leaf-RH 725 (n = 3). (**c**) Derived from modeling silylated primary metabolites of Indian brassica seed versus other 3 organs (n = 3). (**b**) and (**d**) The respective loading S-plots showing the covariance p [1] against the correlation p(cor) [1] of the variables of the discriminating component of the OPLS-DA model. Cut-off values of p < 0.157716 was used. Designated variables are highlighted, and identifications are discussed in the text.

Compared to the unsupervised PCA model, supervised orthogonal partial least squares discriminant analysis (OPLS-DA) was adopted to assess variations among *B. juncea* organs from the two different varieties (Fig. 3) by constructing two respective models of leaf RH-761 against leaf RH-725 (model 1), and seeds against all organs (model 2) from the two different varieties. Leaf model showed higher prediction power with Q^2^ = 0.98 and R^2^ = 0.99 (Fig. 3a) versus seed model at 0.95 and 0.99 (Fig. 3c) with p value < 0.157716 and 1.93251e-010, respectively. Additionally, loading S-plot (Fig. 3b) revealed the abundance of organic acids viz. malic acid, hexanoic acid and sugars viz. fructose, galactose and glucose in leaf RH-725 compared with RH-761. Likewise, loading S-plot (Fig. 3d) revealed that sucrose was abundant only in seeds from the two varieties compared with other organs being rich in monosaccharides viz glucose, fructose, and galactose. The distinct segregation of leaf RH-725 from leaf RH-761 indicated that primary metabolites provided a strong model for differentiation between the two different varieties (RH-725 and RH-761) warranting for studying gene sequencing for explaining differences among the two varieties for a proof of hypothesis.

## Metabolites enrichment analysis

**Figure 4:**
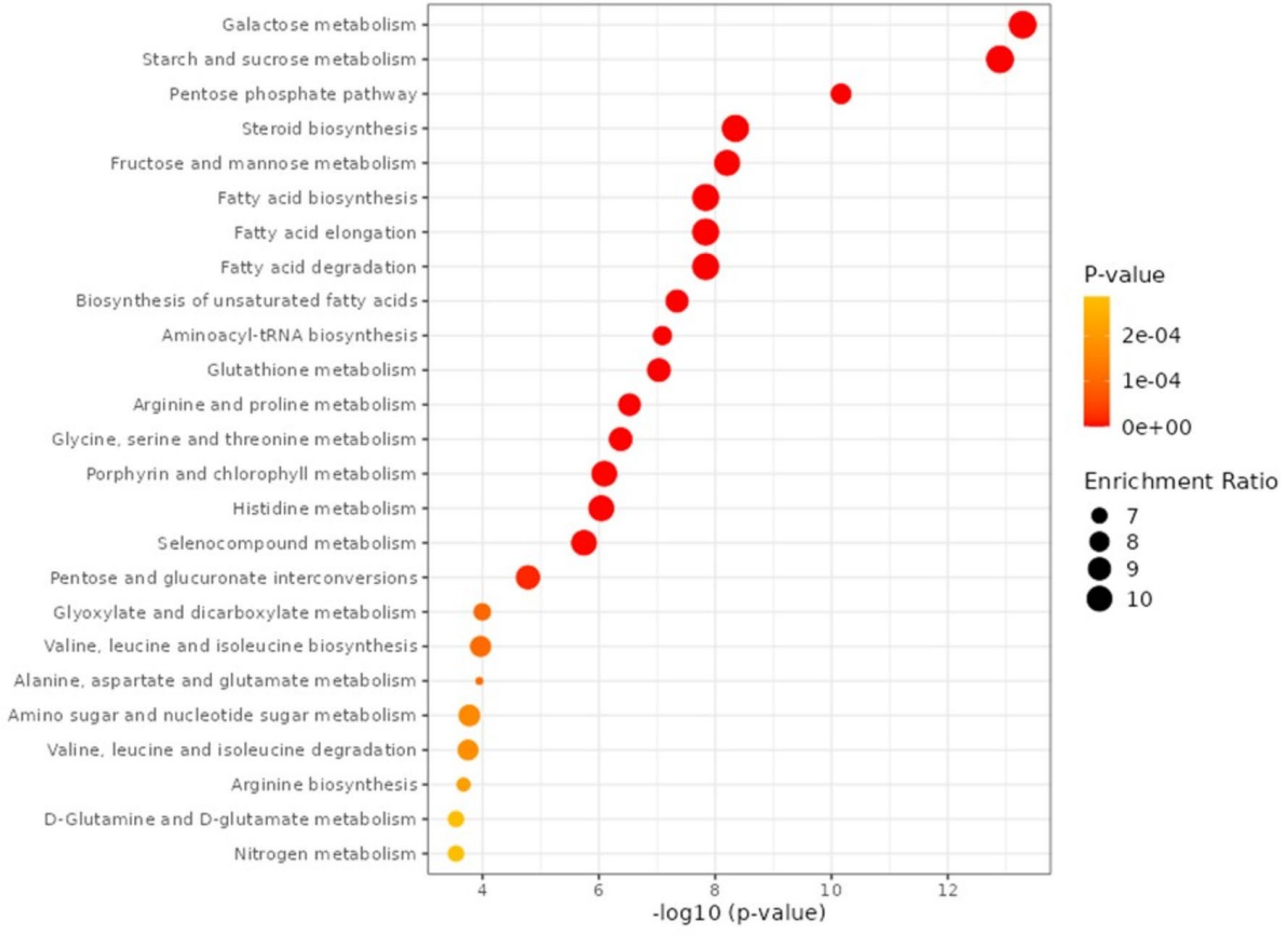
Enrichment analysis of the top 25 metabolites in *Brassica juncea* seeds versus stem and leaf.

**Figure 5:**
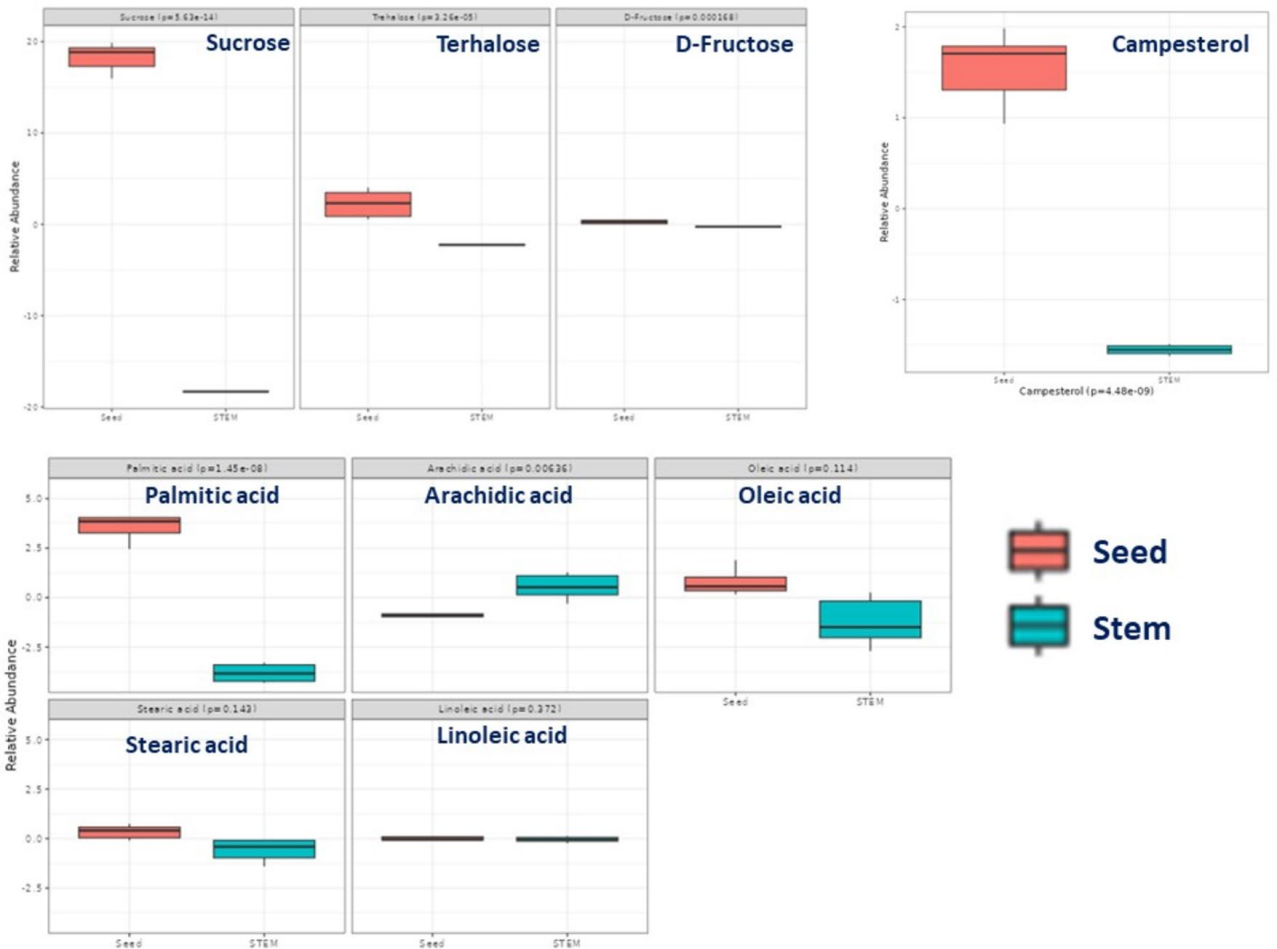
Enrichment analysis of marker metabolites level in *Brassica juncea* seeds versus stem.

Metabolite enrichment analysis of the top 25 metabolites was employed in classification of *B. juncea* seed versus that in stem and leaf (Fig. 4). The top six mapped pathways with the greatest number of differentially expressed genes (DEGs) in the *B. juncea* seed versus stem and leaf group were as follows: galactose metabolism; starch and sucrose metabolism; pentose phosphate; steroids; fructose and mannose metabolism; and fatty acids biosynthesis. Based on the adjusted p-value, metabolites that were enriched in seed versus stem were identified, Fig. 5 revealed for “starch and sucrose metabolism” being significantly enriched in seed (p = 5.63e–14). Additionally, “fatty acid biosynthesis” was significantly enriched in seed represented by palmitic, oleic, and stearic acid. Likewise, steroids biosynthesis was significantly enriched in seed represented by campesterol and as expected considering its lipid rich nature as a storage organ.

## Headspace-SPME–GC–MS volatiles analysis of *B. juncea*

Sulphur metabolites are highly characteristic for brassica crops especially seeds^25^ and contribute for their unique aroma and taste, and further health benefits so they are considered as potential markers to be exploited for *B. juncea* varieties classification. A total of 7 sulphur compounds were identified in two *B. juncea* varieties using headspace–solid-phase microextraction (HS-SPME) coupled to GC/MS targeting seeds aroma profile (Table 2**, **Fig. S1). These sulphur aroma metabolites included pungent thiosulfinates and sulfines are characteristic metabolites in brassica vegetables^12^. 3-Butenyl isothiocyanate was identified as the most abundant compound in *B. juncea* detected at 2.1 and 0.8 ng/mg in RH-725 and RH-761, respectively. In contrast, allyl isothiocyante a major sulphur compound in *Brassica* was detected at higher level in RH-761 than in RH-725 at fold ratio of 1.38. Compared to other previous studies, *B. juncea* is well known for its richness in sulphur compounds^20^. 3-Butenyl isothiocyanate and allyl isothiocyanate were previously identified in *B. juncea* seeds using GC–MS analysis^20^. Sulphur volatiles are abundant in *Brassica* vegetables being derived from the enzymatic hydrolysis of their intact glucosinolates^26^ and has to be further profiled in *B. juncea* using liquid chromatography mass spectrometry (LC/MS) more suited for profiling of the nonvolatile glucosinolates.

**Table 2:** Quantification of sulphur aroma compounds in *Brassica juncea* (ng/mg) analyzed using headspace SPME GC–MS, n = 3.

Peak	Average Rt (min)	Average RI	Metabolites name	Class	RH 725 (ng/mg)	RH 761 (ng/mg)	Fold ratio 761/725
1	2.119	724	Carbon disulfide	Sulphur	0.01 ± 0.00	0.01 ± 0.00	1.26
2	2.718	820	3-Methylcrotononitrile	Sulphur	0.84 ± 0.10	0.35 ± 0.14	0.41
3	3.282	911	Allyl Isothiocyanate	Sulphur	0.70 ± 0.25	0.97 ± 0.34	1.38
4	3.795	994	3-Butenyl isothiocyanate	Sulphur	2.09 ± 0.44	0.82 ± 0.25	0.39
5	3.933	1021	Isothiazole, 5-methyl-	Sulphur	0.01 ± 0.01	0.00 ± 0.00	0.32
6	4.315	1100	Thiazole, 4-ethyl-5-methyl-	Sulphur	0.04 ± 0.00	0.02 ± 0.01	0.51
7	4.583	1154	4,5-Epithiovaleronitrile	Sulphur	0.14 ± 0.08	0.02 ± 0.01	0.16

## Conclusion

Metabolite heterogeneity targeting both nutrients and sulphur aroma compounds of *B. juncea* organs from two different varieties was assessed for the first time using a holistic untargeted GC/MS-based metabolomic approach. 101 Primary metabolites and 7 sulphur compounds were detected in *B. juncea* organs with distinct variations among organs and varieties. Glucose, fructose, and galactose were detected as the most abundant free sugars in *B. juncea* organs, except for seed in which sucrose predominated as the major form, especially in RH-725. Moreover, campesterol and *β*-sitosterol were detected at relatively high levels in seed RH-725 and suggestive that this seed presents a good source of phytosterols, while malic acid was abundant in the leaf from variety RH-725 and serves as a marker for distinguishing it from variety RH-761. Whether the enrichment of malic acid in RH-725 would add to its health benefits and shelf life as a natural preservative should be assessed using bioassays. Multivariate data analysis of nutrients revealed that organs belonging to RH-725 were the most rich in nutrients and posing it more for inclusion in food products or as food additive with stronger health potential. Our study provides evidence on the effect of the cultivation environment on metabolic variations among cultivars in *Brassica* genus. Our study provided the first complementary phytochemical evidence that supports the effect of cultivation environment on metabolic variations among cultivars highlights the nutritional determinants of *B. juncea* organs and highlights RH-725 with improved nutrient composition. Further future studies with an extended approach utilizing other analytical techniques targeting minerals, vitamins, and phytonutrients in *Brassica* to identify the best resources and elucidate the health outcomes of *B. juncea* is recommended.

## Materials and methods

### Plant material & cultivation

*Brassica juncea* L. Czern & Coss. organs including seed, leaf, flower, and stem were collected from two different varieties viz*.* RH 725 and RH 761 grown in Hisar, India during harvest season in 2022 (29.1492° N, 75.7217° E). The collected plant material was lyophilized and kept in the freezer at − 20 °C till analysis using GC–MS. All procedures were conducted in conformity with the pertinent rules and regulations.

### GC–MS analysis of silylated primary metabolites

Freeze dried finely powdered plant part (100 mg) were extracted with 5 mL 100% methanol with sonication for 30 min and frequent vortex shaking. Three different samples from each *B. juncea* organ accession were analyzed under the same conditions to evaluate for biological replicates. Methanol extract (100 µL) was aliquoted in screw-cap vials and left to evaporate under a nitrogen gas stream until complete dryness. For derivatization, 150 µL of N-methyl- *N*-(trimethylsilyl)-trifluoroacetamide (MSTFA) previously diluted 1/1% with anhydrous pyridine was mixed with the dried methanol extract and incubated for 45 min at 60 °C prior to analysis using GC–MS. Separation of silylated derivatives was achieved on a Rtx-5MS (30-m length, 0.25-mm inner diameter and 0.25-m film). Quantitative analysis of primary metabolites followed the detailed protocol previously published in Ref.^1^. Soluble sugars, amino acids, organic acids and fatty acids were quantified using standard curves of glucose, glycine, citric and stearic acids and results were expressed as mg/g. Four serial dilutions were prepared from 10 to 600 ug/mL for establishing the standard curves. Calibration curves for glucose, glycine, citric acid and stearic acids displayed *ca*. 0.99 correlation coefficient.

### SPME–GC–MS volatiles analysis

Freeze dried finely powdered seeds (100 mg) were placed in SPME screw-cap vials (1.5 mL) spiked with 10 µg (*Z*)-3-hexenyl acetate with fibers inserted manually above and placed in an oven kept at 50 °C for 30 min. HS-SPME analysis of the volatile compounds was performed as reported in Ref.^8^ with slight modifications. The fiber was subsequently withdrawn into the needle and then injected manually into the injection port of a gas chromatography–mass spectrometer (GC–MS). GC–MS analysis was adopted on an Agilent 5977B GC/MSD equipped with a DB-5 column (30 m × 0.25 mm i.d. × 0.25µm film thickness; Supelco) and coupled to a quadrupole mass spectrometer. The interface and the injector temperatures were both set at 220 ºC. Volatile elution was carried out using the following gradient temperature program: oven was set at 40 ºC for 3 min, then increased to 180 °C at a rate of 12 °C/min, kept at 180 °C for 5 min, finally increased at a rate of 40 °C/min to 240 °C and kept at this temperature for 5 min. Helium was utilized as a carrier gas with a total flow rate of 0.9 mL/min. For ensuring complete elution of volatiles, SPME fiber was prepared for the next analysis by placing it in the injection port at 220 °C for 2 min. For assessment of biological replicates, three different samples for each accession were analyzed under the same conditions. Blank runs were made during sample analyses. The mass spectrometer was adjusted to EI mode at 70 eV with a scan range set at m/z 40–500.

### Metabolites identification and multivariate data analyses

Identification of both volatile and silylated components was performed by comparing their retention indices (RI) in relation to n-alkanes (C6-C30), mass matching to NIST, WILEY library database and with standards whenever available. Peaks were first deconvoluted using AMDIS software (www.amdis.net) before mass spectral matching. Peak abundance data were exported for multivariate data analysis by extraction using MS-Dial software under same conditions cited in Ref.^27^. Data were then subjected to principal component analysis (PCA), hierarchical clustering analysis (HCA) and partial least squares discriminant analysis (OPLS-DA) using SIMCA-P version 13.0 software package (Umetrics, Umeå, Sweden). Markers were subsequently identified by analyzing S-plot, which was declared with covariance (p) and correlation (pcor). All variables were mean-centered and scaled to Pareto variance. Model validation was assessed by computing the diagnostic indices, viz. Q2 and R2 values, p value and permutation testing.

### Plant ethics

The methods in plant collection and experimentation were carried out in accordance with the IUCN Policy Statement on Research Involving Species at Risk of Extinction and the Convention on the Trade in Endangered Species of Wild Fauna and Flora.

The permissions of the plant collection were obtained in accordance with the guidelines prescribed by the American Society of Plant Taxonomists and adopted by the institutional research committee.

### Supplementary Information

Supplementary Information.

## Data Availability

All data generated or analyzed during this study are included in this published article [and its supplementary information files].
