# Supplementary material for: Comparative metabolome variation in Brassica juncea different organs from two varieties as analyzed using SPME and GCMS techniques coupled to chemometrics
Source: Sci Rep. 2024 Aug 27;14:19900. doi: 10.1038/s41598-024-69865-8 (PMC11350069; doi:10.1038/s41598-024-69865-8)
Supplement: Supplementary file 1 — Supplementary Information. [file 41598_2024_69865_MOESM1_ESM.docx]

**Comparative metabolome variation in Indian mustard (*Brassica juncea*) different organs from two varieties as analyzed using SPME and GC-MS techniques coupled to chemometrics**

**Mohamed A. Farag^1*^,** **Vinod Goyal^2^, Mostafa H. Baky^3^**

*^1^ Pharmacognosy Department, College of Pharmacy, Cairo University, 11562 Cairo, Egypt.*

*^2^ Department of Botany & Plant Physiology, CCS Haryana Agricultural University, Hisar, 125004, Haryana, India*

*^3^ Pharmacognosy Department, Faculty of pharmacy, Egyptian Russian University, Badr city, 11829, Cairo, Egypt.*

***** Corresponding author:

[mohamed.farag@pharma.cu.edu.eg](mailto:mohamed.farag@pharma.cu.edu.eg) (M.A.F.)

Tel.: +011-202-2362245 (M.A.F.)


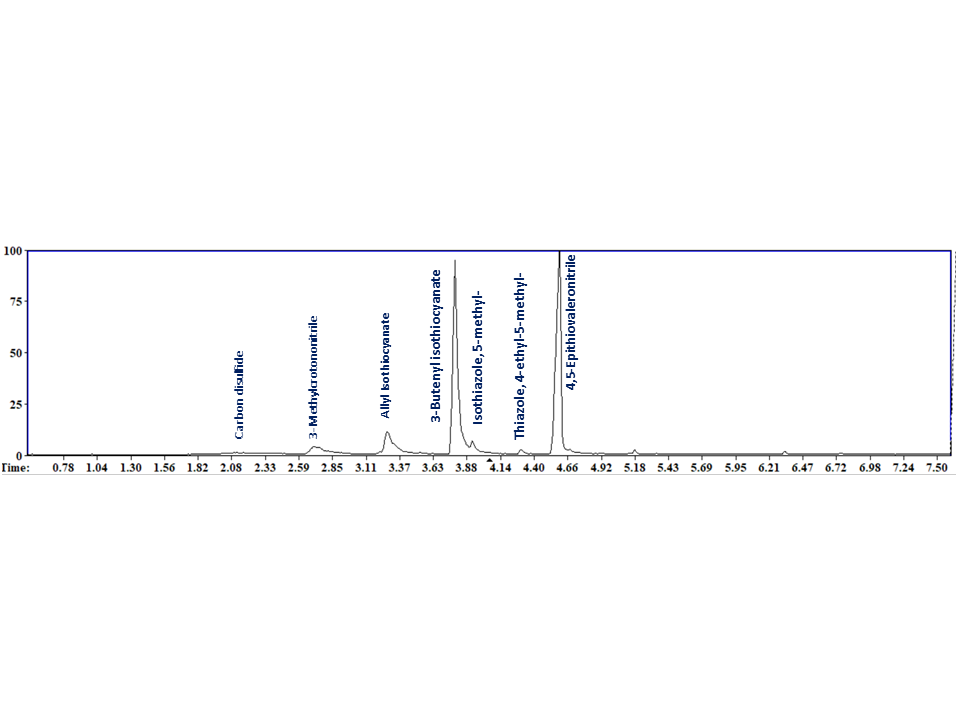


**Figure S1**. Representative GC–MS chromatograms of identified sulfur metabolites in Indian brassica from different varieties.

| **Table S1.** Comparative characteristic parameters between Indian mustard varieties | | |
| --- | --- | --- |
| **Variety** | **RH-725** | **RH-761** |
| Year of release | 2018 | 2019 |
| Yield potential(kg/ha) | 2500-2600 | 2500-2600 |
| Oil content (%) | 40 | 40 |
| Recommended region | Haryana, Punjab, Delhi, Jammu, and Northern Rajasthan | Haryana, Punjab, Delhi, Jammu, and Northern Rajasthan |
| Safety features | TS rainfed | Suitable timely down rainfed condition |
